# Consumption Patterns of Grain-Based Foods among Adults in Canada: Evidence from Canadian Community Health Survey—Nutrition 2015

**DOI:** 10.3390/nu11040784

**Published:** 2019-04-04

**Authors:** Seyed H. Hosseini, Yanni Papanikolaou, Naorin Islam, Patil Rashmi, Arash Shamloo, Hassan Vatanparast

**Affiliations:** 1 College of Pharmacy and Nutrition, University of Saskatchewan, Saskatoon, SK S7N 5A2, Canada; h.hosseini@usask.ca (S.H.H.); naorinislam7@gmail.com (N.I.); rashmi.patil@usask.ca (P.R.); arash.shamloo@usask.ca (A.S.); 2 Nutritional Strategies Inc., Paris, ON N3LOA3, Canada; papanikolaou.yanni@gmail.com

**Keywords:** grains, cluster analysis, consumption patterns, nutrients, socioeconomic status, fortification

## Abstract

In this study, we used the Canadian Community Health Survey-Nutrition (CCHS) 2015 data to examine the consumption patterns of grain-based foods (GBFs) for Canadian adults. We used a *k*-mean cluster analysis based on the contribution of 21 grain-based foods to total energy intake of adults in Canada to find the dietary patterns of GBFs. Cluster analyses rendered seven dietary patterns including: ‘other bread’, ‘cake and cookies’, ‘pasta’, ‘rice’, ‘mixed’, ‘white bread’, and finally ‘whole wheat and whole-grain bread’. ‘No grain’ and ‘rice’ consumers had lower intakes of dietary fibre, folate, iron and calcium, which are the nutrients of public health concern in Canada. Adults consuming a ‘mixed grain’ dietary pattern had a greater daily intake of calcium, potassium, magnesium, riboflavin, and vitamin B6 than those in the ‘no grain’ dietary pattern. We also observed that a considerable proportion of individuals clustered in the ‘rice’ group are immigrants and belong to households with lower income levels.

## 1. Background

Controversies exist about the consumption of grain products due to the association between the intake of refined grain and obesity [1,2]. Nonetheless, the Canadian Community Health Survey (CCHS) cycle 2.2 analyses showed that grain-based foods (GBFs) are important sources of some key nutrients and energy for Canadians [3]. Our team’s analyses (2017) of CCHS-Nutrition 2015 data showed that on average grain products were the source of 45% of folate, 41% of iron, 35% of fibre, and 25.9% of energy consumed daily in Canada. Studies show that the intake of whole grains is associated with lower body weight and lower risk of cardiovascular diseases [4,5,6,7]. Furthermore, other studies, including several meta-analyses, showed the consumption of whole grains is associated with lower rates of colorectal cancer [8], cardiovascular diseases [9,10], type 2 diabetes [11,12], and cancer, as well as lower mortality from all causes [9,13]. In addition, some studies show that higher levels of refined grain consumption are associated with higher prevalence of metabolic syndrome [14] and type 2 diabetes [15]. Therefore, it is suggested that refined grains should be substituted by whole grains in our daily diet [8,13]. However, the mandatory fortification of grain products with folic acid, iron, niacin, riboflavin and thiamine; and voluntary enrichment of these products with magnesium, vitamin B6, calcium and d-pantothenic acid in Canada [16] raises an important question about the value of consuming refined grains along with whole grain products.

Nationally representative data from the 2005–2010 US National Health and Nutrition Examination Surveys (NHANES), indicated that consumption of certain groups of GBFs including bread, ready-to-eat cereals, tortillas and rolls and other grain products was significantly associated with greater intakes of dietary fibre, iron, folate, magnesium, thiamin and niacin in US adults ≥ 19 years-old [17]. Moreover, Papanikolaou and Fulgoni (2017) showed that several GBFs dietary patterns, including pasta, cereals, rice, bread, and mixed grain foods, were associated with better diet quality in adults, as measured by Healthy Eating Index-2010 developed in United States Department of Agriculture (USDA) [18]. Additionally, the researchers found an association between certain grain food consumption patterns and obesity-related outcomes. Specifically, adults ≥19 years-old who consumed pasta, cooked cereals, and rice more often than other grains had significantly lower body weights and smaller waist circumferences. Others have reported that whole grain bread consumption has a somewhat negative effect on body weight or central adipose tissue [19,20].

The analyses of the CCHS cycle 2.2 conducted by our team indicated whole grains contribute to only one-third of Canadians GBFs diets [21]. However, there have been no studies examining the detailed consumption patterns of GBFs and the contribution of these foods to the nutrient intakes of Canadian adults (aged 19 years and older). Furthermore, most existing studies evaluating the association between refund grain and negative health outcomes, did not include enriched refined grains or enriched refined wheat. Thus, the objectives of this study are threefold. First, it aims to identify the consumption patterns of GBFs among Canadian adults. Second, it examines the intakes of some of the key nutrients and several food groups across the identified patterns. Finally, this study evaluates the socioeconomic status (SES) of participants and their Body Mass Index (BMI) based on weight in kilograms devided by their height in metres squared (kg/m^2^) across the consumption patterns of GBFs.

## 2. Subject and Method

### 2.1. Data

We used CCHS-Nutrition 2015 data in this study. It is a cross-sectional survey, which included 24,000 respondents representing Canada’s population. The participants of CCHS-Nutrition 2015 are individuals aged one year and older living in private dwellings in all provinces of Canada. The survey sample does not include those who live on indigenous reserves, residents of institutions, and persons living on military bases. After excluding breastfeeding and pregnant women, and individuals that had uncommon energy intakes of below 200 Kcal or above 8000 Kcal on the first interview day; the sample represents over 27 million Canadians aged 19 years old and over.

### 2.2. Dietary Intake Data

The CCHS-Nutrition 2015 survey includes information about the SES and dietary intake of participants. This information was collected via two inconsecutive 24 h dietary recall interviews based on automated multiple pass method [22]. After the first interview, only about one-third of respondents were interviewed on another day of the week to estimate their usual intakes of foods and nutrients. Food intake data collected in CCHS 2015 is categorized according to Bureau of Nutritional Sciences [23] (BNS) and Tiers [24] categorizations. However, in this study we used the BNS classification to identify patterns of GBFs consumption Canadian adults. The BNS classification considers foods in both the main and recipe levels. Based on BNS classification, GBFs food items were first categorized into 53 groups and then condensed to 21 groups. The contribution of each of the 21 food groups to total individual energy intakes was calculated and used for conducting cluster analysis.

### 2.3. Socioeconomic Status

The SES used in this study include: age, sex, immigration status, ethnicity (white, non-white), household income level (income decile), household education (a member of the household has a higher education degree), location of residence (urban versus rural), food security status (the household is food secure versus the household is food insecure), marital status, smoking status, obesity, BMI, and physical activity. These variables are derived from a set of questions in the CCHS questionnaire.

A person is considered as immigrant if he or she is a landed immigrant in Canada Therefore, it does not include those individuals who live in Canada with refugee status and work or study permit holders. The food security variable, including two categories of food secure and food insecure, was derived from a series of variables that exist in CCHS 2015. In general, food security here refers to “income-relate” food security status. A smoker in our study includes two categories of smokers and non-smokers, where a smoker is an individual who currently smoke either daily or occasionally. BMI is the body mass index defined as the weight divided by the squared value of height. Using BMI, we also defined overweight/obesity variable including two categories of obese/overweight (BMI > 25) and non-obese/overweight (BMI < 25). We also used another variable called active, including two categories of active and non-active. An individual is considered as active if he/she had 150 min or more of moderate or vigorous physical activity per week.

### 2.4. Method

We used *k*-mean cluster analysis [25,26] for the identification of GBFs dietary patterns among Canadian adults. The use of *k*-mean clustering in the identification of dietary patterns is well documented in the literature [27,28]. This method includes an iterative procedure segregating the data into *k* clusters. The process starts with determining *k* random points in the data and observations are allocated to the closest centroid chosen randomly. Afterwards, new averages are calculated for the *k* initial clusters. Then, a new centroid based on the most recent calculated mean is determined. In an iterative procedure, the data is located into the clusters based on their proximity to the calculated centroids.

Determining the optimal number of clusters (i.e., k*) to represent clusters that are distinctive enough is an important phase in the cluster analysis. In many cases, the number of clusters (*k)* is set by the researcher(s) based on existing theories. However, with regards to consumption patterns, such data do not usually exist. Therefore, to determine the optimal number of clusters (k*) we used five approaches described below. Initially, we conducted more than 40 cluster analyses. For the first cluster analysis we set *k* to be equal to two, for the second cluster analysis *k* was equal to three and similarly until the 40th cluster where *k* was 41. Then, in the first approach, we considered the kink appeared in the scree plots of within sum of squares (WSS), the logarithm of WSS, the η^2^ coefficient (η^2^ is a measure that is very similar to the R-squared) and in the case of proportional reduction of error (PRE) [29], we took into account the largest drop in the PRE for each *k.* In these plots, the horizontal axis is the number of clusters ranging from two to 40, and the vertical axis is the measures mentioned above. The second method used is the “cluster stop” command in Stata [30]. In this approach, a measure developed by Caliñski and Harabasz was taken into consideration where for each *k*, the Caliñski and Harabasz measure is calculated and the highest result is used to determine the optimal number of clusters [31]. Because of the high level of sensitivity of cluster analyses to outliers, for each of the 21 food groups, we employed the box plot methods to detect and eliminate the outliers from the dataset.

We used PROC SURVEYREG in SAS 9.2 (SAS Institute, Cary, NC, 2013) to achieve adjusted mean values of nutrients and energy intakes across emerging dietary patterns. Calculating the adjusted mean values, we controlled for age, sex, immigration status, ethnicity (white, non-white), household income level (income decile), household education (a member of the household has a higher education degree), location of residence (urban versus rural), and food security status (the household is food secure versus the household is food insecure). PROC REGRESS and PROC LOGISTIC in SAS 9.2 were used to identify differences between SES of individuals across clusters. We also examined each GBFs consumption pattern across five income levels, where the lowest-income level included households in deciles one and two and the highest income level includes families whose incomes were categorized under deciles nine and 10. The deciles of incomes were adjusted for the number of household members and province of residence.

Following Statistics Canada’s guidelines, due to the complex survey design, the data were weighted and we used bootstrap and sampling weight variables provided by Statistic Canada in the process of conducting analyses so to obtain estimates that can be generalized to regional and national levels [32]. The statistical differences of nutrient intakes and SES across clusters were identified using the overlaps between 95% confidence intervals of the estimates [33]. The individuals with less than one serving of GBFs were considered ‘no grain’ consumers, and their nutrient and energy intake, as well as their SES, were compared with other individuals placed in emerging clusters.

## 3. Results

Our analyses characterized GBFs consumption patterns of over 27 million Canadians that were 19 years old and older. On average, 30.4% of the energy intake of Canadian adults was provided by GBFs. The ‘other bread’, ‘white bread’, ‘cakes and cookies’, ‘whole grain and whole wheat bread’, ‘rice’, ‘pasta’ and ‘whole grain cereals’ were the GBFs that contributed the most to the total energy intakes of Canadian adults, respectively. The food items included in the ‘other bread’ category were bagels, rolls, pita bread, dumplings, croutons, matzo, tortilla, etc.

Cluster analysis resulted in the identification of seven GBFs consumption patterns among Canadian adults consuming grains (i.e., ≥1 serving of grains/day). The dietary patterns were named ‘other bread’; ‘cake and cookies’; ‘pasta’; ‘rice’; ‘mixed pattern’; ‘white bread’, and finally ‘whole grain and whole wheat bread’. The first four GBFs in the mixed cluster and their respective contributions to energy intake were whole grain cereal (14.2%), other bread (10.9%), salty snacks (10.5%), and muffin (9.7%). Other bread in BNS classification includes rolls, bagels, pita bread, matzo, and tortilla. Furthermore, salty snack in here refers to food items such as tortilla chips, crackers, pretzels, etc.

The names assigned to the identified clusters were based on the food group with the highest contribution to total daily energy intake. Table 1 shows the names, weighted percentage, weighted frequency, and the contribution of the first 10 GBFs to total energy intake and energy intake from grain. For instance, rice in the ‘rice’ dietary pattern, which is the primary grain product consumed by 8% of Canadian adults (i.e., 2.2 million individuals), provided about 22% of total energy intake of individuals in this group. For these individuals, rice provides 58% of energy intake from grain products. The second most important grain product in the ‘rice’ cluster is other bread, supplying about 3% of total energy intake of individuals in this cluster. Additionally, it should be noted that about 6.2% of adults in Canada are ‘no grain’ consumers, equivalent to 1.6 million individuals.

‘Whole bread’ and ‘whole grain and whole wheat bread’ were considered as two different groups. According to Health Canada (2013), ‘whole grain bread’ and ‘whole wheat bread’ are two different products; therefore, for the sake of the objectivity of the analysis, we did not merge these two groups, although the food group of ‘whole grain and whole wheat bread’ include both ‘whole grain bread’ and ‘whole wheat bread’.

### 3.1. Nutrient and Energy Intake

**Figure 1:**
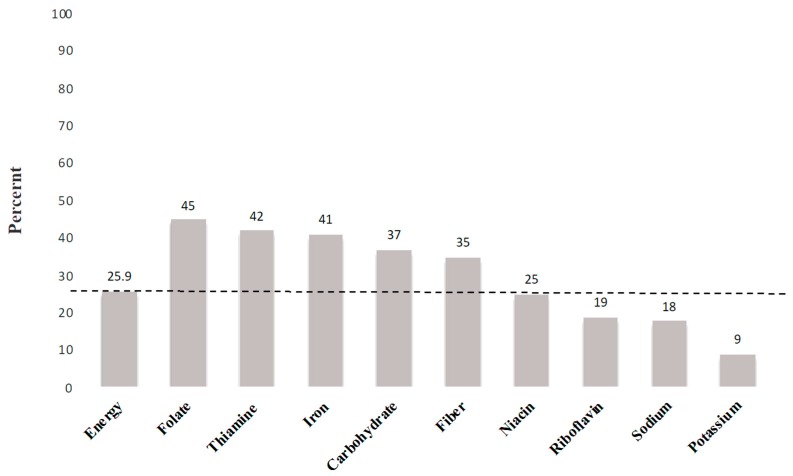
Energy and nutrients from all grain foods in the daily diet of Canadains aged two years old and above. The dashed line represents the percentage of energy provided and identifies nutrients that surpass energy contribution.

Figure 1 shows the average contribution of grains foods to the daily intake of some key nutrients in Canada. On average, more than one-quarter of Canadians’ energy intakes are derived from GBFs (dashed line in Figure 1). GBFs generally supply 37% of daily carbohydrate intakes, 45% of folate, 42% of thiamine, 41% of iron, 35% of fibre, and 25% of niacin intake. Therefore, although GBFs are the main source of energy intake in Canada, they are also the primary source of folate, iron, thiamin, niacin, and fibre. In other words, GBFs are nutrient-rich foods.

Adjusted mean values of total nutrient and energy intakes across identified GBFs and ‘no grain’ consumers are shown in Table 2. The lowest and the highest energy intakes were observed in the ‘no grain’ and the ‘mixed’ clusters, respectively (1457 Kcal/day and 2102.3 Kcal/day). The energy intakes of people in the ‘mixed’ and the ‘cake and cookies’ clusters were significantly higher than all other identified patterns.

With regard to nutrient intakes of public health concern (iron, calcium, vitamin D, and potassium) [34], Canadian adults clustered in the ‘mixed’ group had the highest intake of calcium (914.3 mg/day), while those in the ‘rice’ cluster had the lowest intake of calcium (568.4 mg/day) (Table 2). The first four primary grain products in the ‘mixed’ cluster, comprising about 35% of this pattern portfolio, are whole grain cereal, other bread, salty snacks and muffins. The differences in calcium intakes were not statistically significant across the remaining patterns. It was also observed that the ‘no grain’ consumers had the lowest intake of iron (8.3 mg/day). Excluding the ‘no grain’ group, we did not observe statistically significant disparities in iron intake across identified dietary patterns. However, potassium intake was significantly higher in the ‘mixed’ cluster (3029.7 mg/day).

As shown in Table 2, the ‘no grain’ consumers had significantly lower intakes of folic acid, iron, thiamine, magnesium, and fibre compared to the emerging GBFs dietary patterns. Across the clusters, folic acid was significantly higher than others among the ‘pasta’ consumers. The average intake of dietary fibre was significantly lower in the case of ‘no grains’ consumer compared to all GBFs patterns. In fact, some of the GBFs consumption patterns contributing higher than 6 g dietary fibre per day versus adults avoiding grain foods (‘pasta’: 17.9 ± 0.6; ‘mixed’ grains: 18.6 ± 0.3; ‘whole grain and whole wheat bread’: 18.5 ± 0.5 versus ‘no grains’: 12.4 ± 0.6 g/day).

In addition, the ‘mixed’ pattern was associated with a significantly higher intake of protein, riboflavin, vitamin B6, magnesium, potassium, and calcium. However, individuals clustered in this group also have higher intakes of cholesterol and sugar. Furthermore, sodium intakes are considerably higher among the ‘mixed’ and ‘white bread’ patterns. However, ‘no grain’ and ‘rice’ consumption patterns have the lowest sodium intake.

Table 3 shows the mean intakes of different food group servings consumed in each cluster. On average ‘mixed grain’ and ‘rice’ consumers have considerably higher and lower intakes of ‘milk and alternatives’, respectively. The ‘rice’ consumers have lower intakes of both ‘fluid milk’ and ‘other milk products’ like cheese and butter in comparison with other grain consumption patterns. However, in the case of ‘mixed grain’ consumers, it can be observed that their consumption of ‘other milk’ products is significantly higher than other clusters.

### 3.2. SES and GBFs Patterns

Table 4 shows the differences in SES and BMI across the GBFs dietary patterns. The average BMIs of adults are not different across clusters where examining the overlap of confidence intervals. The age of adults found in the ‘whole wheat and whole grain’ group was significantly higher than other clusters. Furthermore, in contrast to other clusters, an even distribution of males and females was not observed in the ‘no grain’ group where the proportion of females was significantly higher.

Canadian adults who consumed ‘rice’ more than other GBFs were mainly situated in urban areas, a higher proportion of them were married, and a considerable percentage were immigrants. A noteworthy fraction of adults in the ‘white bread’ cluster are members of food insecure households (17%). As it is shown in Table 5, the proportion of Canadian adults with the highest income level was significantly lower in the ‘rice’ cluster, and significantly higher in the ‘mixed’ group.

## 4. Discussion

This first Canadian study, using CCHS-Nutrition 2015 data, involved analysis of GBFs consumption patterns among Canadian adults and intakes of some key nutrients across identified clusters. In addition, this study considered associations of SES factors with GBFs dietary patterns. Our results suggest that consumption of particular GBFs are superior when compared to no grain consumption in the case of vital nutrients provided by GBFs. The intake of iron, folic acid, riboflavin, thiamine, and niacin were higher in specific GBFs consumption patterns compared with ‘no grain’ consumers. These are the mandatory nutrients added to refined grains, implying that the fortification of refined grains is an effective policy. The higher intakes of several nutrients in the grain products made with refined grain justify the importance of a balanced diet including both refined grains and whole-grains. In addition, one can recommend the change in the fortification policy of grain products in Canada where a new policy could include enrichment of whole grain foods. This policy could lead to an increase in both the demand and production of whole grain products. Moreover, examining the consumption patterns of grain products among Canadian adults shows the impacts of SES such as immigration status and income levels on choices of refined grains.

The results of this study indicate GBFs contribute a considerable portion of daily intake of macronutrients and micronutrients. About one-quarter and more than one-third of daily energy intake and carbohydrates intakes, respectively, are supplied by GBFs. Similarly, CCHS cycle 2.2 analysis showed that grains supplied 28.5% of the total daily energy of Canadians adults. However, our analysis put this figure at 25.9%, which could be related to the different data collection methods used in CCHS-2004 and CCHS-2015 [35]. Additionally, our analyses indicated that about 80% of Canadian adults do not consume the recommended number of GBFs servings recommended by the Canada’s Food Guide released in 2007 [21]. If we consider the Canadas’ new food guide suggesting most of the grain intakes should be from whole grain [36] in 2015, a considerable proportion of Canadians had consumption patterns of GBFs that were far from the recommended patterns of grain consumption by Health Canada. Using CCHS 2015 data, we observed that the GBFs are important sources of folic acid, iron, thiamin, and calcium. This observation is likely to be the consequence of the fortification of grain products in Canada [35], which compensates for the loss of nutrients through food processing.

The examination of seven GBFs dietary pattern identified by cluster analysis plus ‘no grain’ pattern of grain the examination of seven GBFs dietary pattern identified by cluster analysis plus ‘no grain’ pattern of grain consumption indicated that almost half of Canadians are found to be in the ‘mixed’ and ‘white bread’ clusters. The ‘mixed’ cluster includes whole grain cereal, other bread (e.g., pita bread, bagel, roll, and matzo), salty snacks, and muffins; foods most likely to be found in breakfast and snack meals of adults in Canada. This cluster also includes sweet and salty snacks, which may explain the significantly higher proportion of daily energy intake by individuals in this cluster. Similarly, regarding the GBFs consumption patterns among US adults, more than 50% of the population were clustered in the ‘yeast bread rolls,’ and ‘mixed grains’, which primarily includes cake and cookies, pies, and salty snacks. Furthermore, in alignment with our results, another study using NHANES data reported the ‘no grain’ cluster had the lowest energy intake [17].

The higher prevalence of immigrant groups in the ‘rice’ cluster may be due to the consistent presence of rice in the Asian, Indian, and South American cuisine [37]. In alignment with previous studies, the higher “starchy staples” were consumed by groups in lower SES categories. Previous studies have indicated that poverty and lower education levels are associated with higher consumption of energy-dense foods, such as those made of refined grains [38,39]. The smaller proportions of adults with the highest level of income in the ‘rice’, cluster is consistent with Bennet’s law, implying an inverse relationship between the rise in income and the demand for “starchy staples” [40]. Bennet ’s law stems from the demand for diversity as a result of having higher levels of income [41]. Additionally, the high proportion of adults with the highest income level in the Mixed cluster is likely to be related to what Timmer (1997) calls the desire for diet diversity.

Although the primary focus of this study is not on the role of diet on body weight, we found no difference in adults’ BMI across the clusters. In contrast, researchers using NHANES data previously observed lower body weights and smaller waist circumferences among individuals predominantly consuming a grain dietary pattern comprised of ‘pasta, cooked cereals, and rice’ compared to a ‘no grain’ dietary cluster [17].

Our study has some limitations. Dietary intake data in CCHS was collected using 24 h recall, which may be subject to under or over-reporting [42]. We initially had 53 GBF groups and then merged them into 21 food groups. The merging of GBF food groups was primarily conducted based on similarities amongst foods in the 53 groups. BNS classification has some limitations in food categories. For instance, the food group called ‘other bread’ includes many grain products that make it difficult to identify the dietary patterns in more detail. Additionally, the inclusion of several products in the ‘other bread’ resulted in this category having the highest contribution to the total energy intake of Canadian adults in our analysis. Furthermore, grain products such as English muffins are classified in more than one category, such as muffins, English muffins, and other bread, leading to double-counting of such products. BNS has two other food groups’ names, ‘whole grain and high fibre cereals’ and ‘other cereals’, which were considered as two food groups because the former group contains both ready-to-eat and cooked breakfast cereals. Therefore, to account for differences of these groups and for tracking the consumption of whole grain cereals versus non-whole grain cereals, we did not collapse these two food groups. Nevertheless, the dietary patterns in this study seem to be distinct enough when they are compared with the results of other studies conducted in the USA [17,43].

## 5. Conclusions

Cluster analysis resulted in the identification of seven distinct GBFs consumption patterns for Canadian adults. In addition, we considered ‘no grain’ (i.e., less than one serving of GBFs) as another pattern of GFBs consumption. Overall, individuals in the ‘no grain’ and ‘rice’ groups had low intakes of some vital nutrients, such as folic acid, iron, calcium, riboflavin, niacin, and thiamine. In addition, our analysis of nutrient intakes among GBF patterns implies that as a result of the mandatory and voluntary enrichment of refined grains, these products may not be considered as inferior foods as they contribute to the intake of nutrients of public health concern and prevent inadequate nutrient intakes among Canadians. Therefore, a balanced diet including both whole and enriched non-whole grains seems to be better than a diet including only whole-grain products. Additionally, our findings showed that GBFs dietary patterns are linked with income level, food insecurity, and immigration status. More detailed investigations are required to conclude the benefit of balanced grain intakes.

## Figures and Tables

**Table 1:** Grain consumption patterns among adults (Canadian Community Health Survey 2015).

GBFs Pattern	The Contribution of GBFs to Total Energy Intake (%)	The Contribution of GBFs Within Cluster (%)	Canadian Adults in the Cluster (%)	Population (*n*)
**Mixed**			**40.7**	**11,000,000**
Whole Grain Cereals	3.2	14.2		
Other Bread	2.5	10.9		
Salty Snacks	2.4	10.5		
Muffin	2.2	9.7		
White Bread	1.6	6.9		
Cakes & Cookies	1.5	6.4		
Whole Bread	1.4	6.2		
Rice	1.2	5.1		
Whole Grain Bread	1.0	4.2		
Whole Grain and Whole Wheat Bread	0.9	3.9		
**White Bread**	**20.5**	**62.3**	**12.2**	**3,300,000**
Cakes & Cookies	1.9	5.6		
Other Bread	1.5	4.6		
Whole Grain Cereals	1.5	4.5		
Pasta	1.2	3.6		
Salty Snacks	1.1	3.4		
Rice	1.0	3.2		
Danish and Doughnuts	0.8	2.4		
Muffin	0.8	2.3		
Other Cereals	0.6	1.9		
**Other Bread**	**23.5**	**63.4**	**10.4**	**2,800,000**
Rice	1.7	4.6		
Whole Grain Cereals	1.7	4.5		
White Bread	1.5	4.0		
Cakes & Cookies	1.4	3.9		
Whole Grain and Whole Wheat Bread	1.2	3.2		
Salty Snacks	1.1	3.0		
Pasta	0.9	2.5		
Muffin	0.9	2.5		
Danish and Doughnuts	0.7	2.0		
**Pasta**	**20.9**	**52.6**	**8.6**	**2,300,000**
White Bread	2.9	7.4		
Other Bread	2.2	5.5		
Whole Grain Cereals	2.1	5.3		
Cakes & Cookies	1.7	4.4		
Salty Snacks	1.5	3.9		
Whole Grain and Whole Wheat Bread	1.5	3.8		
Muffin	1.4	3.4		
Rice	1.2	3.0		
Whole Bread	0.7	1.8		
**Rice**	**21.9**	**58.0**	**8**	**2,200,000**
Other Bread	2.3	6.0		
White Bread	2.2	5.8		
Whole Grain and Whole Wheat Bread	1.9	4.9		
Whole Grain Cereals	1.7	4.6		
Cakes & Cookies	1.6	4.2		
Salty Snacks	1.1	2.8		
Muffin	1.0	2.8		
Pasta	1.0	2.5		
Danish and Doughnuts	0.6	1.6		
Whole Grain and Whole Wheat Bread	0.9	3.9		
**Whole Grain and Whole Wheat Bread**	**17.9**	**56.7**	**8**	**2,200,000**
Whole Grain Cereals	2.4	7.5		
Cakes & Cookies	2.0	6.3		
Other Bread	1.5	4.7		
Rice	1.5	4.6		
Salty Snacks	1.2	3.8		
Pasta	0.9	2.8		
Muffin	0.8	2.6		
White Bread	0.6	1.8		
Other Cereals	0.5	1.6		
**Cakes & Cookies**	**23.5**	**55.8**	**5.9**	**1,600,000**
White Bread	3.5	8.3		
Other Bread	2.8	6.7		
Whole Grain Cereals	2.1	4.9		
Whole Grain and Whole Wheat Bread	1.7	3.9		
Pasta	1.5	3.5		
Rice	1.4	3.3		
Salty Snacks	1.3	3.1		
Muffin	0.9	2.2		
Whole Bread	0.7	1.6		

Whole grain cereals and other cereals were considered as two different groups because whole grain cereals include both cooked cereal and ready-to-eat cereals. GBFs: grain-based foods. The primary GBFs consumed in each cluster are shown with bold font.

**Table 2:** Adjusted Adult Mean (SE) Nutrient and Energy Intakes for All Clusters of GBFs.

	No Grain Consumers	Other Bread	Cakes & Cookies	Pasta	Rice	Mixed	White Bread	Whole Wheat & Whole-Grain Bread	*p*-Value
Energy (Kcal)	1457 (76.8)	1738.6 (39.5)	2040.3 (54.2)	1866.6 (47.02)	1609.8 (44.1)	2102.3 (21.8)	1805.8 (41)	1561.6 (35.7)	0.99
Calcium (mg)	584.1 (31.7)	738.4 (24.6)	768.4 (25)	779.3 (27)	568.4† (21)	914.3 *† (15)	709.02 (20.6)	690.81 (23.3)	0 < 0.0001
Iron (mg)	8.3 *† (0.4)	12.4 (0.3)	13.5 (0.4)	12.9 (0.4)	9.8 (0.29)	13.5 (0.17)	12.7 (0.3)	10.4 (0.3)	0 < 0.0001
Potassium (mg)	2547.8 (83.8)	2438.7 (52.22)	2637.4 (66.5)	2587.1 (65.4)	2337.7 (61.3)	3029.7 *† (34)	2362 (55.4)	2469.5 (56.17)	0 < 0.0001
Folate DFE. (mcg)	263.2 *† (14.6)	461.6 (13.3)	480.7 (19.55)	609.4 *† (17.08)	329.6 (10.8)	455.1 (6.75)	492.5 (12.85)	309.7 (9.36)	0 < 0.0001
Folic Acid (mcg)	23.79 *† (3.49)	122.17 (4.06)	135.61 (8.07)	221.7 *† (7.6)	60.22 (3.51)	103.23 (2.31)	160.2 *† (4.69)	53.83 (2.74)	0 < 0.0001
Riboflavin (mg)	1.62 (0.09)	1.88 (0.05)	1.92 (0.05)	1.92 (0.06)	1.47† (0.04)	2.1 *† (0.03)	1.96 (0.05)	1.61 (0.05)	0 < 0.0001
Thiamin (mg)	0.95 *† (0.04)	1.67 (0.05)	1.69 (0.07)	1.88 (0.06)	1.23 (0.04)	1.69 (0.02)	1.66 (0.04)	1.25 (0.03)	0 < 0.0001
Niacin (mg)	34.55 (2.32)	37.58 (1.05)	37.36 (1.3)	41.48 (1.29)	35.06 (1.18)	43.1 (0.57)	37.02 (0.91)	33.96 (1.01)	0 < 0.0001
Dietary fibres (g)	12.45 *† (0.61)	17.68 (0.57)	17.17 (0.62)	17.89 (0.61)	15.21 (0.63)	18.54 (0.25)	14.59 (0.38)	18.52 (0.48)	0 < 0.0001
Sodium (mg)	1853.5 *† (119.78)	2637.31 (79.46)	2686.9 (91.51)	2674.3 (83.7)	2292 *† (79.3)	2977.33 (41.09)	2941 (76.2)	2607.5 (84.87)	0 < 0.0001
Sugars (g)	73.83 (3.78)	72.32 (1.96)	116.7 *† (3.25)	79.42 (2.59)	65.36 (2.87)	98.3 *† (1.58)	85.52 (2.44)	69.94 (2.21)	0 < 0.0001
Vitamin B6 (mg)	1.74 (0.1)	1.55 (0.04)	1.6 (0.08)	1.55 (0.05)	1.68 (0.05)	1.9 † (0.03)	1.36 *† (0.04)	1.5 (0.05)	0 < 0.0001
Total Carbohydrates (g)	141.9 *† (6.1)	216.5 (4.5)	257.9 *† (7.1)	236.3 (5.7)	212.5 (6.3)	237.5 (2.7)	223.4 (5.1)	189.1 *† (4.5)	0 < 0.0001
Cholesterol (mg)	292.6 (22.8)	238.9 (22.5)	265.4 (10)	237.8 (9.9)	240.8 (10.7)	305.2† (7.1)	254.6 (13.2)	243.9 (12.1)	0 < 0.0001
% Energy from Carbohydrates	40.2 *† (0.89)	50.1 (0.55)	50.4 (0.59)	51.1 (0.55)	53.3 *† (0.6)	45.1 *† (0.27)	49.7 (0.48)	48.7 (0.78)	0 < 0.0001
Saturated Fatty Acid (g)	18.0 (1.2)	20.8 (0.7)	25 (1.0)	21.1 (0.7)	15.0† (0.6)	26.6 (0.4)	22.2 (0.7)	18.1† (0.8)	0 < 0.0001
Fat (g)	57.88 (3.66)	61.59 (1.92)	77.29 (2.67)	64.07 (2.19)	50.2 *† (1.78)	82.05 (1.1)	66.06 (2.05)	56.74 (1.81)	0 < 0.0001
Magnesium (mg)	264.9 (10.84)	283.66 (5.85)	299.92 (8.22)	305.7 (10.11)	281.56 (9.15)	340.2 *† (4)	270.91 (7.28)	308.18 (7.28)	0 < 0.0001
Proteins (g)	74.07 (4.97)	75.59 (2.18)	76.25 (2.3)	78.69 (2.36)	72.24 (2.13)	88.03 *† (1.09)	72.66 (1.74)	69.29 (1.89)	0 < 0.0001
Vitamin A (mcg)	635.37 (49.52)	536.02 (27.29)	661.73 (31.3)	612.16 (27.15)	600.81 (50.47)	745.41 (20.85)	523.96 (18.33)	638.92 (33.41)	0 < 0.0001
Zinc (mg)	8.86 (0.48)	10 (0.3)	10.53 (0.46)	9.92 (0.34)	9.28 (0.28)	11.96 (0.21)	9.43 (0.27)	9.19 (0.29)	0 < 0.0001

* Significantly different than all GBFs patterns (*p* < 0.05). † Significantly different than other emerged clusters (*p* < 0.05). All data are weighted to represent population-level information.

**Table 3:** The average intake of food group servings consumed in identified clusters.

	No Grain Consumers	Other Bread	Cakes & Cookies	Pasta	Rice	Mixed	White Bread	Whole Wheat & Whole-Grain Bread
Total Fruits & Vegetables	5.1 (0.29)	3.8 (0.13)	4.4 (0.21)	4.6 (0.18)	4.3 (0.19)	5 (0.1)	3.8 (0.17)	4.2 (0.16)
Fruits (excluding fruit juice)	1.4 (0.11)	1 (0.06)	1 (0.09)	1.1 (0.08)	1.4 (0.11)	1.5 (0.05)	1.1 (0.08)	1.3 (0.11)
Dark Green Vegetables	0.7 (0.08)	0.4 (0.04)	0.5 (0.06)	0.6 (0.08)	0.8 (0.08)	0.6 (0.03)	0.3 † (0.03)	0.6 (0.06)
Orange Vegetables	0.3 (0.05)	0.2 (0.04)	0.2 (0.04)	0.2 (0.02)	0.2 (0.03)	0.2 (0.02)	0.1 (0.02)	0.2 (0.03)
Potato	0.8 (0.09)	0.5 (0.05)	0.7 (0.1)	0.3 (0.04)	0.2 (0.03)	0.8 (0.04)	0.7 (0.08)	0.5 (0.05)
Other Vegetables	1.6 (0.21)	1.1 (0.06)	1.1 (0.1)	1.8† (0.08)	1.2 (0.08)	1.4 (0.04)	1.1 (0.06)	1 (0.07)
Total Milk & Alternatives	1 (0.08)	1.3 (0.07)	1.3 (0.08)	1.4 (0.07)	0.8 *† (0.07)	1.7 *† (0.05)	1.3 (0.06)	1.2 (0.07)
Fluid Milk or Soy Milk	0.5 (0.05)	0.5 (0.03)	0.6 (0.05)	0.7 (0.06)	0.4† (0.04)	0.7 (0.03)	0.5 (0.04)	0.5 (0.03)
Other Product Made of Milk	0.6 (0.05)	0.8 (0.05)	0.7 (0.06)	0.8 (0.06)	0.4† * (0.05)	1†* (0.03)	0.8 (0.05)	0.7 (0.06)
Total Meat & Alternatives	2.4 (0.28)	1.9 (0.1)	1.9 (0.1)	1.7 (0.09)	2 (0.08)	2.4† (0.05)	1.9 (0.08)	1.9 (0.08)
Poultry	1.1 (0.27)	0.7 (0.09)	0.5 (0.09)	0.5 (0.07)	0.7 (0.07)	0.7 (0.03)	0.4 (0.04)	0.5 (0.04)
Beef	0.4 (0.05)	0.4 (0.04)	0.5 (0.09)	0.4 (0.04)	0.3 (0.03)	0.5 (0.03)	0.4 (0.04)	0.3 (0.03)
Legumes	0.2 (0.04)	0.2 (0.02)	0.2 (0.04)	0.3 (0.04)	0.3 (0.04)	0.4 (0.02)	0.3 (0.03)	0.3 (0.04)
Egg	0.3 (0.03)	0.2 (0.05)	0.2 (0.02)	0.2 (0.02)	0.2 (0.03)	0.2 (0.01)	0.3 (0.03)	0.3 (0.03)
Processed Meat	0.1 (0.02)	0.3 (0.03)	0.2 (0.03)	0.2 (0.02)	0.1 (0.02)	0.3 (0.02)	0.4 (0.03)	0.3 (0.03)

The unit of measure in here is the number of serving consumed. * Significantly different than all GBFs patterns (*p* < 0.05). † Significantly different than other emerged clusters (*p* < 0.05). All data are weighted to represent population-level information.

**Table 4:** Differences in scioeconomic and body mass index across clusters, age group 19 years old and above.

SES	No Grain Consumers	Other Bread	Cakes and Cookies	Pasta	Rice	Mixed	White Bread	Whole Wheat & Whole Grain Bread
Mean age (SEM)	47.9 (1)	46.5 (0.7)	51 (1.5)	44.9 (1)	46.3 (0.8)	49.5 (0.4)	51.9 (0.7)	55.8 *† (0.9)
BMI (SEM)	27.6 (0.6)	27.1 (0.4)	26.8 (0.4)	26.7 (0.3)	26.5 (0.4)	27.5 (0.2)	27.9 (0.3)	28.1 (0.4)
% male	40 *†	47	55	50	53	51	51	47
% Caucasian	76	70	74	73	31 *†	83	82	78
Smoker	26	16	14	20	15	18	28†	12
Education (% university grad) ^1^	38	42	33	41	47	40	29	32
Married (%)	58	66	65	63	73 *†	64	62	63
Food secure (%)	87	89	87	92	88	90	83 *†	90
Urban (%)	80	84	84	85	93 *†	81	81	78
Immigrant (%)	26	35	32	26	67 *†	20	21	28
Overweight/obese (%)	61	57	60	58	53	64	67	69
Physical Act (%)	68	73	69	73	72	76	66	73
Mean age (SEM)								55.8 *† (0.9)

* Significantly different than all GBFs patterns (*p* < 0.05). † Significantly different than other emerged clusters (*p* < 0.05). ^1^ A member of the household has a higher education degree. All data are weighted to represent population-level information. SES: socioeconomic status; SEM: standard error of the mean.

**Table 5:** Income distribution and GBFs dietary patterns.

	The Lowest	Low	Middle	High	The Highest
No Grain Consumers	19.4	14.1 *	20.6	20.2	25.7
Other Bread	21.2	18.1	20.3	19.1	21.3
Cakes and Cookies	21.0	21.6	18.9	21.3	17.2
Pasta	16.5	23.3	20.3	18.3	21.6
Rice	28.6	24.7	17.9	17.2	11.6 *
Mixed	16.4	18.0	19.4	21.2	25.0 *
White Bread	25.8	23.3	16.4	16.1	18.3
Whole Wheat & Whole-Grain Bread	22.2	20.2	20.8	19.3	17.5

* Significantly different than all GBFs patterns (*p* < 0.05). All data are weighted to represent population-level information. The unit of the figure in this table is percentage.
